# Endophthalmitis after open globe injuries: changes in microbiological spectrum and isolate susceptibility patterns over 14 years

**DOI:** 10.1186/1869-5760-4-5

**Published:** 2014-02-18

**Authors:** Animesh Jindal, Avinash Pathengay, Kopal Mithal, Subhadra Jalali, Annie Mathai, Rajeev Reddy Pappuru, Raja Narayanan, Jay Chhablani, Swapna R Motukupally, Savitri Sharma, Taraprasad Das, Harry W Flynn

**Affiliations:** 1 L V Prasad Eye Institute, GMR Varalakshmi Campus, Visakhapatnam 530040, India; 2 Srimati Kannuri Santhamma Centre for Vitreoretinal Diseases, L V Prasad Eye Institute, KAR Campus, Hyderabad 500034, India; 3 L V Prasad Eye Institute, Bhubaneswar Eye Institute, Bhubaneswar, Odisha 751024, India; 4 Jhaveri Microbiology Centre, L V Prasad Eye Institute, KAR Campus, Hyderabad 500034, India; 5 Department of Ophthalmology, Bascom Palmer Eye Institute, Miller School of Medicine, University of Miami, Miami, FL 33124, USA

**Keywords:** Open globe injury, Endophthalmitis, Microbiology

## Abstract

**Background:**

The objective of this study was to evaluate the microbiologic spectrum and antimicrobial susceptibility of isolates in post-traumatic endophthalmitis and compare with our earlier published report. A retrospective review was conducted on 581 consecutive patients with culture-proven post-traumatic endophthalmitis at L. V. Prasad Eye Institute, India, from January 2006 to March 2013.

**Findings:**

A total of 620 isolates from 581 patients were identified (565 bacteria and 55 fungi). The most common isolate was *Bacillus* spp. (106/620, 17.1%) closely followed by *Streptococcus pneumoniae* (105/620, 16.9%), and coagulase-negative *Staphylococci* (97/620, 15.6%). In our earlier report, the commonest bacteria included *Streptococcus* spp. (30/139, 21.6%) and gram-positive coagulase-negative micrococci (26/139, 18.7%). Gram-positive isolates were usually susceptible to vancomycin (98.2%). Gram-negative isolates were generally susceptible to gatifloxacin (92.9%), ofloxacin (89.4%), chloramphenicol (88.6%, *Pseudomonas* isolates were often resistant), amikacin (83.5%), and ceftazidime (77.2%). Fourteen years ago, the most sensitive antibiotic was ciprofloxacin for both gram-positive bacteria (95.12%) and gram-negative bacteria (100%).

**Conclusions:**

The microbiological spectrum of post-traumatic endophthalmitis has remained unchanged over the last 14 years, and *Bacillus* spp. continues as the most common infecting organism. Vancomycin is the drug of choice for empiric coverage of gram-positive bacteria. Susceptibility of gram-negative bacteria to commonly used antimicrobials (amikacin and ciprofloxacin) has decreased by 10% - 15% and to ceftazidime has increased by 10.5%.

## Findings

### Background

Open globe injuries (OGI) are a common cause of infectious endophthalmitis. The incidence of endophthalmitis in this setting varies from 3% to 48% in various studies, depending on a variety of factors which include setting of trauma, delay of repair, retained intraocular foreign body, and involvement of the crystalline lens [1]. Post-traumatic endophthalmitis is either a community-acquired infection from the unsterile material causing trauma or caused by the inoculation of the normal ocular flora at the time of injury. The purpose of the study is to describe the microbial spectrum and antimicrobial susceptibility in cases of endophthalmitis after OGI and to compare the results with the earlier published data from the same center [2].

### Methods

This was a retrospective, non-comparative, consecutive case series. Microbiology records were reviewed of all the culture-proven endophthalmitis cases after OGI treated at L. V. Prasad Eye Institute, Hyderabad, India, between January 2006 and March 2013. Bacterial isolates were identified using Analytical Profile Index (API, Bio Meriux, France). The isolate susceptibility to various antimicrobials was determined by the Kirby-Bauer disk diffusion method. Susceptibilities for fungal isolates were not performed. The study was approved by the Institutional Review Board of L. V. Prasad Eye Institute and adhered to the guidelines of the Declaration of Helsinki.

### Results

A total of 620 isolates from 581 samples were identified. A total of 38 samples (6.5%) had polymicrobial infection; 37 samples grew two isolates, and one sample grew three organisms. The isolates included gram-positive cocci (296, 47.7%), gram-positive bacilli (117, 18.8%), gram-negative bacteria (142, 22.9%), fungi (55, 8.87%), and *Corynebacterium* (10, 1.61%) (Table 1).

**Table 1:** Microbiological spectrum and antimicrobial susceptibilities in patients with endophthalmitis following open globe injuries

**Organism**	**Total/percentage(previous study)**^**2**^	**Total/percentage (current study)**	**Amikacin**			**Cefazolin**			**Ceftazidime**			**Chloramphenicol**			**Gentamicin**			**Gatifloxacin**			**Ciprofloxacin**			**Moxifloxacin**			**Vancomycin**			**Ofloxacin**		
			***n***	***S***	**%**	***n***	***S***	**%**	***n***	***S***	**%**	***n***	***S***	**%**	***n***	***S***	**%**	***n***	***S***	**%**	***n***	***S***	**%**	***n***	***S***	**%**	***n***	***S***	**%**	***n***	***S***	**%**
Gram-positive cocci	63/45.3	296/47.7	274	155	56.5	294	266	90.4	137	79	57.6	295	284	96.2	295	217	73.5	292	263	90.1	296	219	73.9	239	206	86.2	289	284	98.2	295	240	81.4
Coagulase-negative *Staphylococcus*	25/18	97/15.6	86	81	94.2	96	90	93.7	41	18	43.9	96	93	96.9	97	89	91.7	97	96	98.9	97	75	77.3	75	69	92	95	93	97.9	97	76	78.4
*Staphylococcus aureus*	5/3.6	11/1.8	11	11	100	11	11	100	5	3	60	11	11	100	11	10	90.9	11	10	90.9	11	5	45.4	10	8	80	11	11	100	11	6	54.5
*Streptococcus* sp.	30/21.6	172/27.7	163	52	31.9	172	154	89.5	81	54	66.6	172	166	96.5	172	108	62.7	169	143	84.6	172	127	73.8	143	119	83.2	168	165	98.2	171	143	83.6
Other gram-positive cocci	3/2.2	16/2.6	14	11	78.6	15	11	73.3	10	4	40.0	16	14	87.5	15	10	66.7	15	14	93.3	16	12	75.0	11	10	90.9	15	15	100	16	15	93.7
Gram-positive bacilli	24/17.3	117/18.8	114	109	95.6	115	63	54.7	75	12	16	117	109	93.1	117	112	95.7	117	116	99.1	116	108	93.1	101	99	98.0	114	109	95.6	117	116	99.1
*Bacillus* sp.	20/14.4	106/17.1	103	101	98.1	104	54	51.9	67	8	11.9	106	98	92.4	106	105	99.1	106	105	99.0	106	101	95.2	94	92	97.9	103	98	95.1	106	106	100
Other GPB	4/2.9	11/1.8	11	8	72.7	11	9	81.8	8	4	50	11	11	100	11	7	63.6	11	11	100	10	7	70	7	7	100	11	11	100	11	10	90.9
Gram negative organism	25/18	142/25	140	117	83.5	16	7	43.7	136	105	77.2	141	125	88.6	142	125	88.0	141	131	92.9	142	123	86.6	116	96	82.7	16	4	25	142	127	89.4
*Pseudomonas* sp.	10/7.2	16/2.6	16	12	75	1	0	0	16	11	68.7	16	5	31.2	16	12	75	16	12	75.0	16	11	68.7	13	8	61.5	1	0	0	16	11	68.7
*Enterobacter sp*.	2/1.4	25/4.0	25	22	88	5	2	40	23	21	91.3	25	24	96	24	23	95.8	25	24	96	25	24	96	19	19	100	5	2	40	25	24	96
*E. coli*	4/2.9	14/2.3	14	12	85.7	-	-	-	14	12	85.7	14	13	92.8	14	13	92.8	14	11	78.6	14	11	78.6	12	7	58.3	-	-	-	14	10	71.4
*Haemophilus sp*.	-	14/2.3	14	7	50	-	-	-	13	5	38.5	14	14	100	14	11	78.6	14	14	100	14	12	85.7	13	11	84.6	-	-	-	14	14	100
Other gram-negative organisms	9/6.5	73/11.8	71	64	90.1	10	5	50	70	56	80	72	69	95.8	74	66	89.2	72	70	97.2	73	65	89	59	51	86.4	10	2	20	73	68	93.2
*Corynebacterium*	5/3.6	10/1.6	5	4	80	10	9	90	3	0	0	10	9	90	10	9	90	10	9	90.0	10	7	70	8	6	75.0	10	10	100	10	9	90
Total bacteria	119/85.6	565/91.1	533	385	72.2	435	345	79.3	351	196	55.8	563	527	93.6	564	463	82.0	560	519	92.7	564	457	81.1	464	407	87.7	429	407	94.8	564	492	87.2
Total fungi	20/14.4	55/8.9	Not done																													
*Aspergillus*	9/6.5	20/3.2																														
*Acremonium*	1/0.7	1/0.32																														
*Bipolaris*	1/0.7	2/0.32																														
*Cladosporium*	1/0.7	2/0.32																														
*Candida*	-	3/0.48																														
*Fusarium*	2/1.4	1/0.16																														

All isolates were not tested for susceptibility to all antibiotics leading to ‘*n*’ being less than the total isolates. GPB, gram-positive bacilli; *n*, number of isolates tested for antibiotic susceptibility; *S*, number of isolates sensitive to the antibiotic.

The most common organism isolated was *Bacillus* spp. (106/620, 17.1%) followed by *Streptococcus pneumoniae* (105/620, 16.9%) and coagulase-negative *Staphylococci* (97/620, 15.6%). *Enterobacter* was the most common gram-negative isolate (25 of 142 gram-negative isolates). *Aspergillus* spp. was the most common fungus (20 of 55 fungal isolates) (Table 1).

The spectrum of the isolates identified in this series was similar to that reported in 1999 from the same center [2], with coagulase-negative *Staphylococci* (CoNS), *Streptococcus*, and *Bacilllus* sp. being the three most common organisms (Table 1). The only differences in the spectrum of organisms were the relatively lower incidence of fungal endophthalmitis in the current study (current 8.9% vs earlier 14.4%) and higher incidence of gram-negative isolates (current 25% vs earlier 18%). But, both of these were not statistically significant (*p* = 0.257 and 0.058, respectively).

Both gram-positive cocci and gram-positive bacilli were generally susceptible to vancomycin (98.2% and 95.6%, respectively). *Bacillus* sp. was also susceptible to vancomycin (95.1%) as well as to gentamicin (99.1%), amikacin (98.1%), chloramphenicol (92.4%), ciprofloxacin (95.2%), ofloxacin (100%), gatifloxacin (99.1%), and moxifloxacin (97.9%) but its susceptibility to ceftazidime was very poor (11.9%). Gram-negative bacteria were generally susceptible to gatifloxacin (92.9%) followed by ofloxacin (89.4%), chloramphenicol (88.6%), ciprofloxacin (86.6%), amikacin (83.5%), and ceftazidime (77.2%). Among the fluoroquinolones, both gram-positive and gram-negative organisms were most susceptible to gatifloxacin (92.7% and 92.9%, respectively). The antimicrobial susceptibilities of the various isolates are detailed in the Table 1.

### Discussion

The susceptibility of gram-positive organisms continues to be highest to vancomycin. Susceptibility of CoNS to ciprofloxacin had reduced from 100% in 1999 report [2] to 77.3% in the current report (*p* = 0.007). Similar fluoroquinolone susceptibility trend towards CoNS was noted in the report by Schimel et al. [3]. Susceptibility of gram-negative organisms has slightly decreased over the last two decades. In the 1999 report, gram-negative organisms were 100% susceptible to ciprofloxacin and 95% to amikacin [2]. This has now reduced to 86.6% and 83.5%, respectively, but this change was statistically not significant (*p* = 0.078 and *p* = 0.206, respectively). The susceptibility of gram-negative isolates to ceftazidime has increased from 66.7% in 1999 [2] to 77.2% in the current study, and this difference is also not significant (*p* > 0.05).

The current study indicates that the microbiological spectrum in post-traumatic endophthalmitis has remained the same over the last two decades. Intravitreal vancomycin remains the drug of choice for empiric coverage of gram-positive bacteria. Susceptibility of gram-negative bacteria to commonly used antimicrobials including amikacin and ciprofloxacin has decreased by 10% to 15% and to ceftazidime has increased by 10.5% as compared to the past two decades though statistically not significant. Thus, intravitreal amikacin can be used for empiric coverage of gram-negative bacteria. Ofloxacin is a good option as a systemic antibiotic for broad-spectrum empiric coverage. Also, we report a high prevalence of *Bacillus* endophthalmitis in open globe injuries that are known to be highly virulent with rapid progression to panophthalmitis [4]. Similar high prevalence of *Bacillus* endophthalmitis following open globe injuries has been reported in the literature [5,6].

### Conclusions

The microbiological spectrum remains similar over the past 14 years. The susceptibility of gram-negative organisms to commonly used antibiotics has decreased over the time period. Empiric treatment of post-traumatic endophthalmitis with broad-spectrum, combination antibiotics based on the susceptibility pattern in that region is very important for successful anatomical and visual outcomes.

## Competing interests

The authors declare that they have no competing interests.

## Authors’ contributions

AJ carried out the data collection and data analysis and drafted the manuscript. AP is one of the treating physicians and also carried out the correction of the manuscript. KM, SJ, AM, RRP, RN, JC, and TD are the other treating physicians. SRM and SS are the microbiologists. TD and HWFJ corrected the manuscript. All authors read and approved the final manuscript.
